# AtPIP1;4 and AtPIP2;4 Cooperatively Mediate H_2_O_2_ Transport to Regulate Plant Growth and Disease Resistance

**DOI:** 10.3390/plants13071018

**Published:** 2024-04-03

**Authors:** Xiaohui Yao, Yanjie Mu, Liyuan Zhang, Lei Chen, Shenshen Zou, Xiaochen Chen, Kai Lu, Hansong Dong

**Affiliations:** 1 National Key Laboratory of Wheat Improvement, College of Plant Protection, Shandong Agricultural University, Taian 271018, China; 2 Qingdao King Agroot Crop Science, Qingdao 266071, China

**Keywords:** aquaporin, H_2_O_2_ transport, plant growth, immunity

## Abstract

The rapid production of hydrogen peroxide (H_2_O_2_) is a hallmark of plants’ successful recognition of pathogen infection and plays a crucial role in innate immune signaling. Aquaporins (AQPs) are membrane channels that facilitate the transport of small molecular compounds across cell membranes. In plants, AQPs from the plasma membrane intrinsic protein (PIP) family are utilized for the transport of H_2_O_2_, thereby regulating various biological processes. Plants contain two PIP families, PIP1s and PIP2s. However, the specific functions and relationships between these subfamilies in plant growth and immunity remain largely unknown. In this study, we explore the synergistic role of AtPIP1;4 and AtPIP2;4 in regulating plant growth and disease resistance in Arabidopsis. We found that in plant cells treated with H_2_O_2_, AtPIP1;4 and AtPIP2;4 act as facilitators of H_2_O_2_ across membranes and the translocation of externally applied H_2_O_2_ from the apoplast to the cytoplasm. Moreover, AtPIP1;4 and AtPIP2;4 collaborate to transport bacterial pathogens and flg22-induced apoplastic H_2_O_2_ into the cytoplasm, leading to increased callose deposition and enhanced defense gene expression to strengthen immunity. These findings suggest that AtPIP1;4 and AtPIP2;4 cooperatively mediate H_2_O_2_ transport to regulate plant growth and immunity.

## 1. Introduction

Plants have developed sophisticated signaling and response systems to adapt to various biotic and abiotic stresses. Reactive oxygen species (ROS), such as H_2_O_2_, play a crucial role in these processes by acting as signaling molecules that regulate plant growth, development, stress responses, and defenses [1,2]. Rapid production of ROS, especially in apoplasts, indicates effective plant recognition of pathogens [2,3,4]. The plasma membrane-localized NADPH oxidase (NOX) is the primary site of the pathogen-associated molecular pattern (PAMP)-induced ROS burst in plants, which catalyzes the production of superoxide (O_2_^−^) by transferring electrons from cytoplasmic NADPH to the plasma ectodomain, and finally the production of H_2_O_2_ by superoxide dismutase [4,5,6]. H_2_O_2_ can enter the plant cytoplasm via AQPs. Subsequently, H_2_O_2_ serves as a signaling to cross-talk with plant immunity pathways, such as activating pathogen-associated molecular pattern-triggered immunity (PTI) and systemic acquired resistance (SAR), enhancing the plants resistance to future pathogen attacks [2,7,8,9,10,11].

AQPs are integral membrane proteins initially characterized as water (H_2_O) transport channels [12]. Subsequent studies have revealed that AQPs can also transport more than 20 small molecule compounds, such as CO_2_ [11,13,14,15], H_2_O_2_ [9,10,11], NH_3_ [16], NO [17], and others [18,19]. Through their function in mediating substrate transport, AQPs regulate a variety of pathological and physiological processes [4,20,21,22]. AQP4, a key protein in astrocytes, plays a crucial role in the development and regulation of water balance in the brain and spinal cord by facilitating bidirectional water movement across cell membranes [23]. In mice, AQPs are essential for the transport of H_2_O_2_ into colonic epithelial cells, playing pivotal roles in wound repair, defense against infection, and inflammation in the colon [24]. In plants, PIPs transport small solutes to regulate plant growth and defense [21,22]. For instance, AtPIP1;4 facilitates the transport of H_2_O_2_ from the apoplast into the cytoplasm, thereby activating the SAR and PTI pathways in response to bacterial pathogens [9]. AtPIP2;1 is crucial for the intracellular accumulation of H_2_O_2_ following treatment with flg22 or ABA, thereby promoting stomatal closure [8]. In rice, OsPIP2;2 transports pathogen-induced H_2_O_2_ into the cytoplasm and facilitates the translocation of the OsmaMYB into the nucleus, intensifying defense responses against pathogens [10]. OsPIP1;3 and OsPIP2;1 enhances rice growth and grain yield by facilitating CO_2_ transport [15,25]. ZmPIP2;5 facilitates maize growth by transporting H_2_O [26,27,28]. TaPIP2;10 enhances both wheat growth and defense by promoting CO_2_ transport for photosynthesis and facilitating the cellular uptake of H_2_O_2_ to increase resistance against pathogen and aphid infections [11,29].

Plant PIPs can be categorized into two families based on sequence similarity: PIP1 and PIP2 [21,30,31]. The main structural distinctions between PIP1 and PIP2 lie in the lengths of their N-segment and C-terminal, and they also exhibit differences in their functional properties [32]. Several studies have indicated that certain PIP1 proteins have low efficiency in H_2_O transport [33,34], but they often acts as solute channels [15,35,36]. In comparison, the PIP2 group has been found to possess a higher capacity for H_2_O transport [37,38]. Some studies have reported that PIP1 is unable to localize to the plasma membrane (PM) when expressed alone, and must be co-expressed with PIP2 to correctly localize in the PM [39,40]. Many studies have focused on how PIP1 or PIP2 family proteins regulate plant physiology and pathology, respectively [9,10,11,25,27,29,38]. However, the specific contribution of PIP1 and PIP2 family proteins in H_2_O_2_ transport remain unclear, and their potential in regulating plant growth and immunity is still unknown.

This study bridges this knowledge gap by confirming that AtPIP1;4 and AtPIP2;4 cooperatively mediate H_2_O_2_ transport to regulate plant growth and immunity. We demonstrate that both AtPIP1;4 and AtPIP2;4 act as cooperative contributors to plant growth. Furthermore, we present evidence that AtPIP1;4 and AtPIP2;4 are concomitant channels for H_2_O_2_ transport from apoplast to cytoplasm, thereby activating PTI to enhance plant disease resistance.

## 2. Results

### 2.1. Both AtPIP1;4 and AtPIP2;4 Contribute to Plant Growth

**Figure 1:**
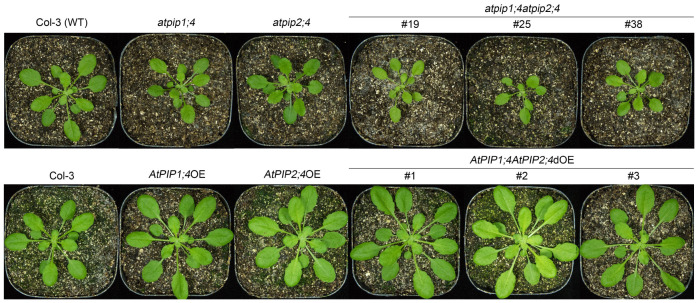
Both AtPIP1;4 and AtPIP2;4 contribute to plant growth. Photographs were taken of 3-week-old Arabidopsis plants during their growth.

To investigate the functional relationship between AtPIP1;4 and AtPIP2;4, we generated the *atpip1;4* and *atpip2;4* double mutant (*atpip1;4atpip2;4*) Arabidopsis lines by hybridizing the single mutants produced previously [9,41]. To further explore this relationship, we also generated Arabidopsis lines with the double overexpression of *AtPIP1;4* and *AtPIP2;4* genes (*AtPIP1;4AtPIP2;4*dOE) by crossing the single gene overexpression lines (*AtPIP1;4*OE and *AtPIP2;4*OE). Three representative homogeneous double mutant lines (*atpip1;4atpip2;4* #19, #25, and #38) and three homogeneous double overexpression lines (*AtPIP1;4AtPIP2;4*dOE #1, #2, and #3) were obtained (Figure 1). To assess the contribution of AtPIP1;4 and AtPIP2;4 to plant growth, we evaluated the growth of rosette leaves in Arabidopsis plants. The growth of the plants was observed and photographed at 3, 4, and 6 weeks after sowing. Compared with wild-type plants (WT), rosette leaf growth was reduced in both *atpip1;4* and *atpip2;4* plants. Furthermore, the leaf growth of *atpip1;4atpip2;4* #19, #25, and #38 were even more severely inhibited than that of the single mutants (Figure 1 and Figure S1). In contrast, the rosette leaves of AtPIP1;4OE and AtPIP2;4OE were larger than wild-type plants, respectively. Similarly, *AtPIP1;4AtPIP2;4*dOE plants had larger leaves than *AtPIP1;4*OE and *AtPIP2;4*OE (Figure 1 and Figure S1).

**Figure 2:**
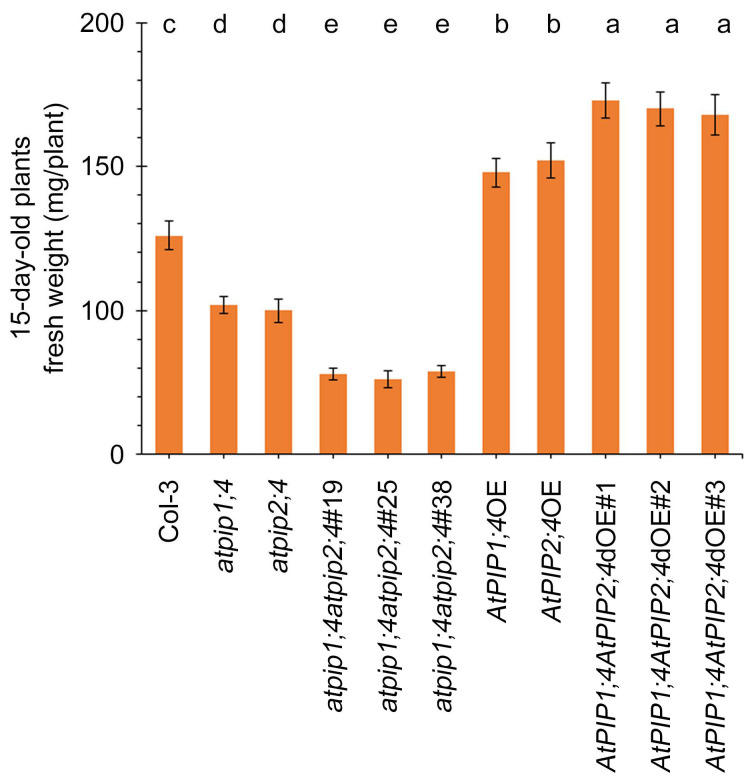
AtPIP1;4 and AtPIP2;4 contribute to plant fresh weight. The fresh weight of 15-day-old plants. Data are shown as means ± SEM (*n* = 6). Lowercase letters indicate significant differences by one-way ANOVA and Duncan’s multiple range tests (at *p* ≤ 0.01).

To further elucidate the relationship between AtPIP1;4 and AtPIP2;4 in regulating plant growth, we measured the fresh weight of 15-day-old plants. Compared with the WT plants, the fresh weight of *atpip1;4* and *atpip2;4* mutants significantly decreased, and significantly reduced further in *atpip1;4atpip2;4* (Figure 2). Conversely, the fresh weights of *AtPIP1;4*OE and *AtPIP2;4*OE were significantly higher at 15 days (Figure 2). Additionally, the fresh weights of *AtPIP1;4AtPIP2;4*dOE showed an even more significant increase. These results indicate a synergistic effect on plant growth attributed to AtPIP1;4 and AtPIP2;4 in Arabidopsis.

### 2.2. AtPIP1;4 and AtPIP2;4 Are Concomitant Channels for H_2_O_2_ Transport from Apoplast to Cytoplasm

To clarify how AtPIP1;4 and AtPIP2;4 function in transporting H_2_O_2_, we assayed the translocation of externally applied H_2_O_2_ to the cytoplasm. H_2_O_2_ was applied externally to plant leaves, and H_2_O_2_ content in the apoplast and cytoplasm were monitored by using the H_2_O_2_-specific fluorescent probe. We utilized the H_2_O_2_ probes 2′,7′-dichlorofluorescin (H_2_DCF) and Amplex Red (AR), which are able to penetrate the membranes and react with cytoplasmic H_2_O_2_ to produce intense fluorescence, thus enabling the detection of cytoplasmic H_2_O_2_ in living cells [9,42]. Additionally, the Amplex Ultra Red (AUR) probe cannot penetrate the membranes; therefore, it is employed to detect apoplastic H_2_O_2_ [42]. These probes have been widely used to detect the translocation of H_2_O_2_ [9,10,11,29,43].

**Figure 3:**
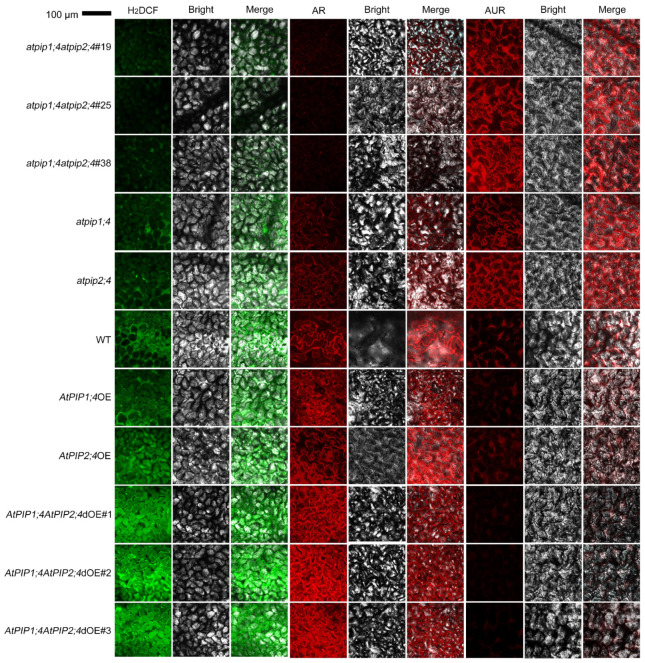
AtPIP1;4 and AtPIP2;4 collaborate to transport H_2_O_2_ from apoplast to cytoplasm in Arabidopsis. LSCM images showing H_2_DCF- and AUR-probed apoplastic H_2_O_2_ and AR-probed cytoplasmic H_2_O_2_ in leaves of the Arabidopsis. The plants, 45 min before LSCM, had been treated with 0.2 mM H_2_O_2_.

We examined the intracellular accumulation of H_2_O_2_ in leaves of different plant types, including the WT, *atpip1;4*, *atpip2;4*, *atpip1;4atpip2;4*, *AtPIP1;4*OE, *AtPIP2;4*OE, and *AtPIP1;4AtPIP2;4*dOE plants. These plants were externally treated with an aqueous solution of 0.2 mM of H_2_O_2_ and pure water as a control (Figure 3 and Figure S3). After 45 min, apoplast and cytoplasm H_2_O_2_ were monitored using confocal laser scanning microscopy (CLSM). In WT plants, the majority of externally applied H_2_O_2_ enters the cytoplasm, while a small portion remains in the apoplast. Compared with the WT plants, *atpip1;4* and *atpip2;4* mutants showed a significant decrease in cytoplasmic H_2_O_2_ content and a significant increase in apoplastic H_2_O_2_ content. In the double mutant *atpip1;4atpip2;4*, there was a notable increase in H_2_O_2_ content in the apoplast, while a significant decrease in cytoplasmic H_2_O_2_ was observed, compared to the WT plants or the single mutants *atpip1;4* or *atpip2;4*. Conversely, the cytoplasmic H_2_O_2_ in *AtPIP1;4*OE and *AtPIP2;4*OE were significantly higher than in the WT plants. Moreover, the *AtPIP1;4AtPIP2;4*dOE exhibited an even higher concentration of cytoplasmic H_2_O_2_ compared to *AtPIP1;4*OE and *AtPIP2;4*OE. In contrast, compared to WT plants, *AtPIP1;4*OE and *AtPIP2;4*OE showed markedly reduced apoplastic H_2_O_2_ content, with even lower levels observed in *AtPIP1;4AtPIP2;4*dOE (Figure 3). In control groups treated with H_2_O, all plant types displayed very low levels of H_2_O_2_ in their leaves (Figure S2).

**Figure 4:**
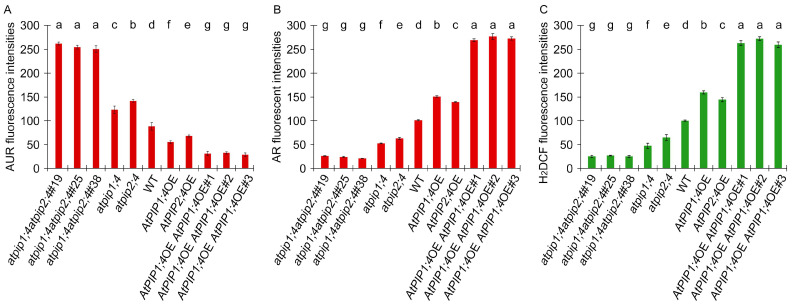
AtPIP1;4 and AtPIP2;4 govern H_2_O_2_ translocation in plants. (**A**–**C**) The plants, 45 min before LSCM, had been treated with 0.2 mM H_2_O_2_. The fluorescence density was quantitatively analyzed using the ImageJ software (ImageJ-v1.8.0.112). (**A**) Assessment of apoplastic H_2_O_2_ content detected by AUR. (**B**) Assessment of cytoplasmic H_2_O_2_ content detected by AR. (**C**) Assessment of cytoplasmic H_2_O_2_ content detected by H_2_DCF. (**A**–**C**) Data are shown as means ± SD (*n* = 9). Lowercase letters indicate significant differences by one-way ANOVA and Duncan’s multiple range tests (*p* < 0.05).

We quantify the fluorescence density, and used this parameter to evaluate the cytoplasmic H_2_O_2_ content detected by AR and H_2_DCF (Figure 4B,C), the apoplastic H_2_O_2_ content detected by AUR (Figure 4A). The differences in apoplastic and cytoplasmic H_2_O_2_ content in different plants are significant. Compared to WT plants, the loss-of-function mutants *atpip1;4* and *atpip2;4* exhibited an increase of 28% and 37% in apoplastic H_2_O_2_ content, while their cytoplasmic H_2_O_2_ contents decreased by 48% and 38%, respectively (Figure 4A,B). Conversely, the overexpression of *AtPIP1;4* and *AtPIP2;4* led to a decrease in apoplastic H_2_O_2_ content by 38% and 23%, and an increase in cytoplasmic H_2_O_2_ content by 32% and 27%, respectively (Figure 4A,B). Further investigation revealed that in comparison with WT plants, the apoplastic H_2_O_2_ content in the *atpip1;4atpip2;4*#19, *atpip1;4atpip2;4*#25, and *atpip1;4atpip2;4*#38 mutants increased by 66%, 65%, and 65%, respectively (Figure 4A), compared with WT plants, while their cytoplasmic H_2_O_2_ content decreased by 74%, 77%, and 79% (Figure 4B). In contrast, the apoplastic H_2_O_2_ content in *AtPIP1;4AtPIP2;4*dOE#1, *AtPIP1;4AtPIP2;4*dOE#2, and *AtPIP1;4AtPIP2;4*dOE#3 were reduced by 65%, 59%, and 67%, respectively (Figure 4A), compared with WT plants, while their cytoplasmic H_2_O_2_ content increased by 62%, 63%, and 63%, respectively (Figure 4B). These results indicate that both AtPIP1;4 and AtPIP2;4 can mediate H_2_O_2_ transport, with AtPIP1;4 having a stronger transport capacity.

**Figure 5:**
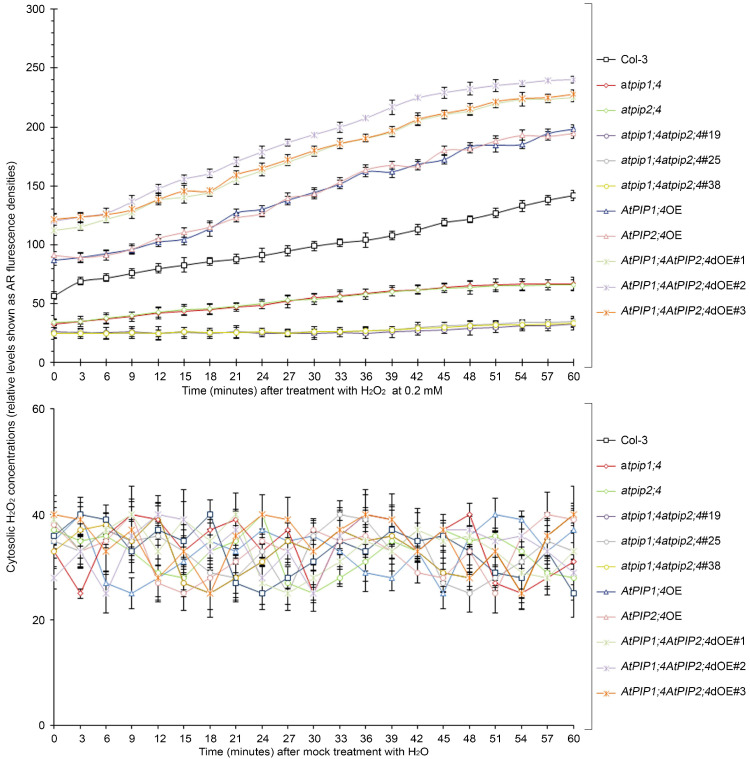
AtPIP1;4 and AtPIP2;4 are concomitant channels for H_2_O_2_ transport. Chronological changes in the H_2_O_2_-probing AR fluorescence densities in leaves of 2-week-old plant seedlings after 0.2 Mm H_2_O_2_ or H_2_O treatment (means ± SEM, *n* = 9).

To analyze the dynamics of H_2_O_2_ transport, plant leaves were treated with 0.2 mM H_2_O_2_ or H_2_O, and the cytoplasmic H_2_O_2_ concentration was quantified by a microplate reader (Figure 5). Within 60 min after the application of H_2_O_2_, a large amount of H_2_O_2_ quickly entered the cytoplasm of plants overexpressing single or both *AtPIP1;4* and *AtPIP2;4* genes. However, the translocation of H_2_O_2_ was significantly reduced in the *AtPIP1;4* and *AtPIP2;4* single or double mutant plants. At 60 min, compared to the WT plants, the cytoplasmic H_2_O_2_ levels increased by 39.6% and 37.3% in *AtPIP1;4*OE and *AtPIP2;4*OE, respectively. Meanwhile, the cytoplasmic H_2_O_2_ levels increased by 58.7%, 69.2%, and 60.4% in *AtPIP1;4AtPIP2;4*dOE#1, *AtPIP1;4AtPIP2;4*dOE#2, *AtPIP1;4AtPIP2;4*dOE#3 plants, respectively. In contrast, the cytoplasmic H_2_O_2_ levels decreased by 76.8%, 75.4%, and 76.4% in *atpip1;4atpip2;4*#19, *atpip1;4atpip2;4*#25, and *atpip1;4atpip2;4*#38 plants, respectively (Figure 5). Plants treated with water showed no significant difference in translocated H_2_O_2_ within 60 min (Figure 5). In addition, we detected the superoxide dismutase (SOD) activity in all plants, confirming that the increase in cytoplasmic H_2_O_2_ levels was a result of externally applied H_2_O_2_ translocation (Figure S3). These results suggest that AtPIP1;4 and AtPIP2;4 both facilitate the translocation of H_2_O_2_ from the apoplast to the cytoplasm, and they are concomitant channels for H_2_O_2_ transport.

### 2.3. AtPIP1;4 and AtPIP2;4 Synergize in Mediating the Cytosolic Import of Apoplastic H_2_O_2_ Induce by Bacterial Infection

**Figure 6:**
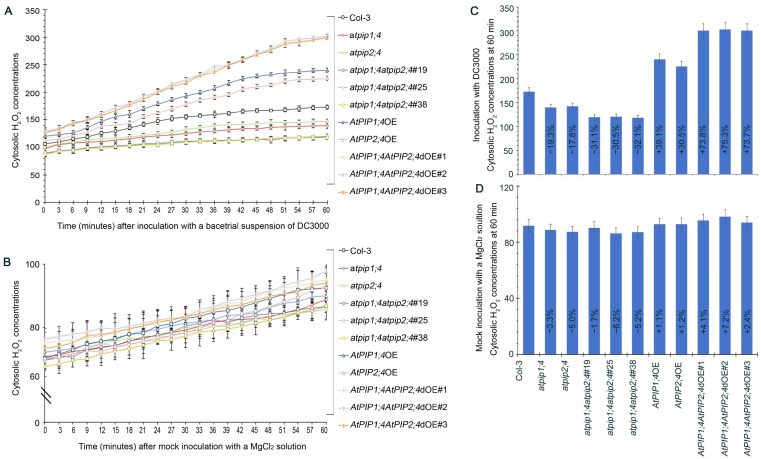
AtPIP1;4 and AtPIP2;4 synergize in the transport of H_2_O_2_ induced by bacterial infection. (**A**) Chronological changes in the H_2_O_2_-probing AR fluorescence densities in leaves of 2-week-old plant seedlings after DC3000 treatment. (**B**) Chronological changes in the H_2_O_2_-probing AR fluorescence densities in leaves of 2-week-old plant seedlings after MgCl_2_ solution treatment. (**C**,**D**) Inoculation with DC3000 or MgCl_2_ and the cytosolic H_2_O_2_ accumulation at 60 min. The percentages shown in the graph represent the relative values of each plant line compared to the wild-type. (**A**–**D**) Data are shown as means ± SEM (*n* = 9).

DC3000, a *Pseudomonas syringae* pv. tomato strain known for its pathogenicity on Arabidopsis thaliana, was used for the bacterial inoculation experiments. This strain is widely utilized in plant–pathogen interaction studies due to its ability to elicit strong immune responses in Arabidopsis, making it an ideal candidate for our investigation into H_2_O_2_ transport and plant immunity [9,44]. To clarify whether AtPIP1;4 and AtPIP2;4 transport H_2_O_2_ in the process of plant resistance to bacterial infection, plant leaves were inoculated with DC3000 for leaf infiltration, and the accumulation of cytoplasmic H_2_O_2_ within 60 min was monitored with an AR probe. Compared with mock infiltration (inoculation with MgCl_2_ solution) (Figure 6B,D), DC3000 significantly induced the accumulation of cytoplasmic H_2_O_2_ in leaves (Figure 6A,C). The superoxide dismutase (SOD) activity was similar in all plant leaves, confirming that the increase in cytoplasmic H_2_O_2_ was indeed caused by the apoplastic H_2_O_2_ translocation induced by DC3000 (Figure S4).

Starting from 15 min, the cytoplasmic H_2_O_2_ content in *AtPIP1;4*OE, *AtPIP2;4*OE, and *AtPIP1;4AtPIP2;4*dOE plants were significantly higher than in WT plants, whereas the cytoplasmic H_2_O_2_ accumulation in *AtPIP1;4* and *AtPIP2;4* single or double gene mutant plants were limited (Figure 6A). At 60 min, the cytoplasmic H_2_O_2_ content in *AtPIP1;4*OE and *AtPIP2;4*OE plants were 1.39-fold and 1.31-fold higher than in WT plants, respectively. The average H_2_O_2_ content in *AtPIP1;4AtPIP2;4*dOE plants was 1.74 times that of WT plants. Meanwhile, compared to WT plants, the cytoplasmic H_2_O_2_ content in *atpip1;4* and *atpip2;4* decreased by 19.3% and 17.8%, respectively. This reduction was further pronounced in *atpip1;4atpip2;4*, where the cytosolic H_2_O_2_ content was 31.2% lower than in WT plants (Figure 6C). These results suggest that AtPIP1;4 and AtPIP2;4 synergistically mediate the cytosolic import of apoplastic H_2_O_2_ induced by bacterial infection.

### 2.4. AtPIP1;4 and AtPIP2;4 Synergize in Enhancing Plant Resistance to Bacterial Infection

**Figure 7:**
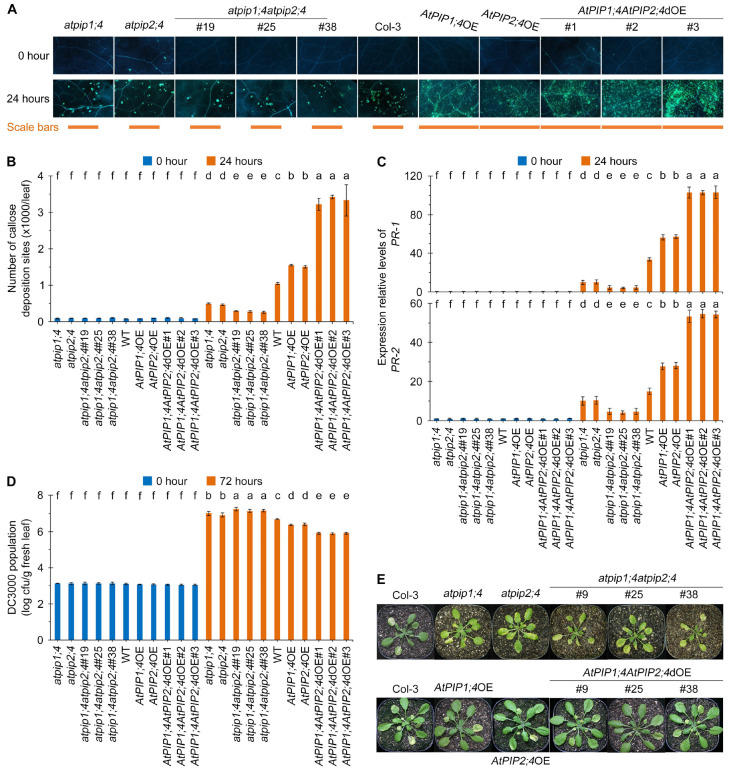
AtPIP1;4 and AtPIP2;4 synergize in enhancing plant resistance to bacterial infection. (**A**) DC3000-induced callose deposition in Arabidopsis. The leaves of 3-week-old plant seedlings were treated with DC3000 for 0 h or 24 h and then stained by aniline blue. (**B**) Quantification of callose deposition from (**A**). Data are shown as means ± SEMs (*n* = 6). (**C**) Expression levels of pathogenesis-related genes *PR1* and *PR2* in plants inoculated with DC3000 for 24 h. (**D**,**E**) Evaluation of *Pst* DC3000 virulence based on bacterial populations in leaves 3 days after inoculation (dai) (**D**) and on leaf symptoms (**E**) at 9 dai. Data are shown as means ± SEMs (*n* = 6). (**B**–**D**) Lowercase letters indicate significant differences by one-way ANOVA and Duncan’s multiple range tests (*p* < 0.05).

H_2_O_2_ serves as a critical signaling molecule in plant immune responses. We have confirmed that AtPIP1;4 and AtPIP2;4 synergistically facilitate the entry of H_2_O_2_ into the cytoplasm from the apoplast (Figure 4, Figure 5 and Figure 6), which led us to investigate their contribution to plant immunity. Callose, a widely distributed β-1,3-glucan in plants, and is closely related to H_2_O_2_ and has become a popular model system for quantifying plant immunity [9,10,11,45,46]. After 24 h of DC3000 treatment, all plants exhibited callose deposition (Figure 7A). Among them, the callose deposition in *AtPIP1;4AtPIP2;4*dOE plants was the highest, averaging 3.05 times that of the WT plants. The callose deposition in *AtPIP1;4*OE and *AtPIP2;4*OE plants was 1.42 and 1.39 times that of the WT plants, respectively. Conversely, callose deposition in *atpip1;4* and *atpip2;4* mutants were limited to 46.3% and 43.0% of the WT plants, respectively. Furthermore, *atpip1;4atpip2;4* displayed the lowest callose deposition, averaging only 25.9% of WT plants (Figure 7A,B).

To clarify how AtPIP1;4 and AtPIP2;4 enhance plant immunity, we also detected the expression of the basic defense genes *PR1* and *PR2* after DC3000 treatment (Figure 7C,D). Compared with WT plants, both *AtPIP1;4*OE and *AtPIP2;4*OE showed significantly upregulated expression of *PR1* and *PR2* after bacterial infection. Moreover, the *AtPIP1;4AtPIP2;4*dOE plants had an even higher level of defense gene expression compared to *AtPIP1;4*OE and *AtPIP2;4*OE plants. Conversely, compared with WT plants, the expression of defense genes in *atpip1;4* and *atpip2;4* plants were significantly inhibited. Further investigation revealed that the expression of *PR1* and *PR2* in *atpip1;4atpip2;4* plants was the lowest (Figure 7C,D).

We assessed the contribution of AtPIP1;4 and AtPIP2;4 to pathogenic bacterial resistance. Compared with WT plants, *atpip1;4* and *atpip2;4* mutants showed higher DC3000 bacterial proliferation, and the leaf discoloration and disease severity of *atpip1;4atpip2;4* mutant plants were significantly increased (Figure 7D,E). Statistical analysis indicated that the DC3000 bacterial population and leaf spot disease severity was significantly lower in *AtPIP1;4*OE and *AtPIP2;4*OE plants compared to WT plants. Notably, the *AtPIP1;4AtPIP2;4*dOE had the least bacterial population and leaf spot disease among all tested plants (Figure 7D,E). In other words, plant resistance to pathogenic bacteria was weakened by *AtPIP1;4* and *AtPIP2;4* mutations, strengthened by *AtPIP1;4* and *AtPIP2;4* overexpression, and further weakened and strengthened by the double mutants and double overexpression. These results suggest that AtPIP1;4 and AtPIP2;4 synergize in enhancing plant resistance to bacterial infection.

### 2.5. AtPIP1;4 and AtPIP2;4 Synergize in Intensifying PTI

**Figure 8:**
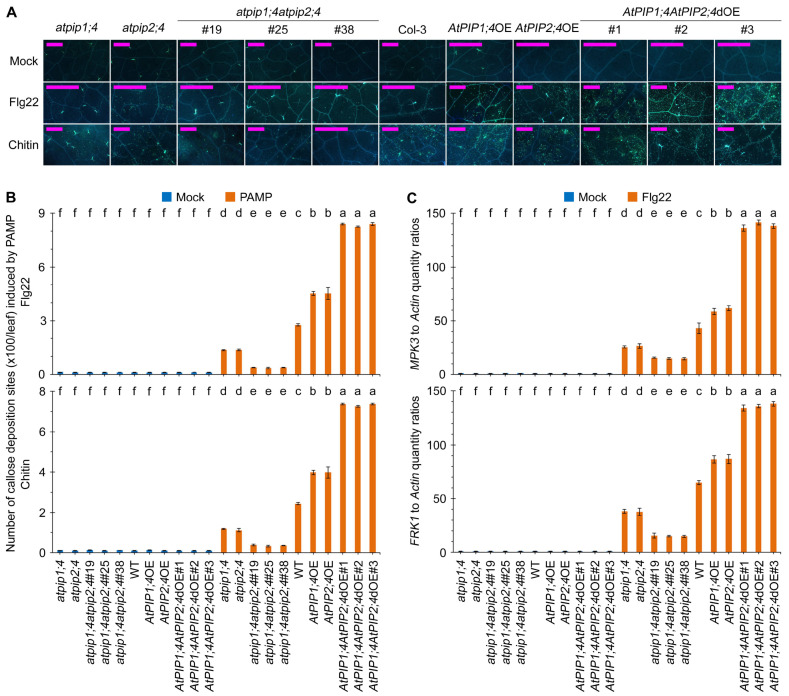
AtPIP1;4 and AtPIP2;4 synergize in intensifying PTI. (**A**) Flg22- and chitin- induced callose deposition in Arabidopsis. The leaves of 3-week-old plant seedlings were treated with 10 μM flg22 and 10 μM chitin for 16 hours and then stained by aniline blue. (**B**) Quantification of callose deposition from (**A**). Data are shown as means ± SEM (*n* = 6). (**C**) Expression levels of PTI-related genes *MPK3* and *FRK1* expression levels in plants treated with flg22 for 60 min; plants were treated with water (mock), an aqueous solution of 10 μM flg22 and then used in the qRT-PCR assays. Data are shown as means ± SEM (*n* = 6). (**B**,**C**) Lowercase letters indicate significant differences by one-way ANOVA and Duncan’s multiple range tests (*p* < 0.05).

To investigate the effects of AtPIP1;4 and AtPIP2;4 on plant immunity, we first measured the callose deposition in leaves of WT, *atpip1;4*, *atpip2;4*, *atpip1;4atpip2;4*, *AtPIP1;4*OE, *AtPIP2;4*OE, and *AtPIP1;4AtPIP2;4*dOE plants treated with flg22 and Chitin (well-known fungal pathogen-associated molecular pattern, PAMP). Callose deposition at the site of plant cell wall infection is a typical PTI response, which can mitigate the invasion of pathogens [45,47]. Compared with WT, the callose deposition in *atpip1;4* and *atpip2;4* leaves decreased by 50.7% and 50.1%, respectively, while the callose deposition in *atpip1;4atpip2;4*#19, *atpip1;4atpip*2;4#25, and *atpip1;4atpip2;4*#38 plant leaves decreased by 86.0%, 87.5%, and 86.1%, respectively, after 24 h of treatment with 10 μM flg22. In contrast, compared with WT, the callose deposition in *AtPIP1;4*OE and *AtPIP2;4*OE leaves increased by 63.8% and 64.2%, respectively. The callose deposition in leaves of *AtPIP1;4AtPIP2;4*dOE plants further increased, with a 203.3%, 198.5%, and 203.9% increase in callose deposition in *AtPIP1;4AtPIP2;4*dOE#1, *AtPIP1;4AtPIP2;4*dOE#2, and *AtPIP1;4AtPIP2;4*dOE#3 leaves, respectively (Figure 8A,B). Similar results were obtained with treatment of 0.1 mg/mL chitin; compared with WT, callose deposition increased due to the overexpression of *AtPIP1;4* and *AtPIP2;4*, and decreased due to the functional loss of *AtPIP1;4* and *AtPIP2;4.* Furthermore, the callose deposition further increased or decreased due to the double overexpression or functional loss of *AtPIP1;4* and *AtPIP2;4* (Figure 8A,B).

Flg22 and chitin activate the expression of *MPK3* and *FRK1*, which serve as positive regulators of the PTI defense response [9,44,48]. Both PAMPs can effectively induce the expression of *MPK3* and *FRK1* in WT plants. Compared with WT plants, the induction of *MPK3* and *FRK1* expression by two PAMPs was significantly suppressed in *atpip1;4* and *atpip2;4* plants, with further inhibition observed in *atpip1;4atpip2;4* plants (Figure 8C,D and Figure S5). Conversely, compared with WT plants, the expression levels of *MPK3* and *FRK1* are significantly elevated in *AtPIP1;4*OE and *AtPIP2;4*OE, and further increased in *AtPIP1;4AtPIP2;4*dOE plants after 24 h of flg22 and chitin treatment (Figure 8C,D and Figure S5). These results indicate that AtPIP1;4 and AtPIP2;4 synergistically regulate the PTI pathway.

## 3. Discussion

AQPs were initially defined as water transport channels, but it has been demonstrated that they can also mediate the transport of many other substrates [12,14,18]. H_2_O_2_ is an important immune signaling molecule, and members of the PIP family in plants have been proven to mediate H_2_O_2_ transport [8,9,10,11]. In plants, PIP family is divided into PIP1 and PIP2 subfamilies based on the conservation of their sequences, with 13 members comprising the PIP family in Arabidopsis [49,50]. Our previous research has shown that members of the Arabidopsis PIP1 family, such as AtPIP1;4, can enhance photosynthesis by promoting CO_2_ transport, and they can facilitate the transport of apoplastic H_2_O_2_ generated by pathogens or induced by flg22 into the cytoplasm, thereby enhancing plant disease resistance [9,51]. However, AtPIP1;4 may not be the sole facilitator of H_2_O_2_ transmembrane transport; it is possible that members of the PIP2 also play a synergistic role [9,41]. This is an important topic for exploring whether PIP1 and PIP2 redundantly coordinate the transport of H_2_O_2_ to regulate plant growth and immunity.

Our study finds that AtPIP1;4 and AtPIP2;4 regulate plant growth, which is similar to earlier reports [51,52]. Some plant PIPs have been proven to be efficient CO_2_ transport channels, capable of promoting CO_2_ transport to enhance photosynthesis, thereby facilitating growth and grain yield [11,15,25]. We have also identified the important role of AtPIP1;4 in plant growth in our previous research, where AtPIP1;4 interacts with the Hpa1 to enhance CO_2_ transport, net photosynthetic rate, and mesophyll conductance, thereby promoting plant growth [51]. However, it is still unclear whether other PIPs cooperate with AtPIP1;4 to regulate plant growth and disease resistance. This study explores the functions of AtPIP1;4 and AtPIP2;4 in these processes. We examined not only the independent roles of AtPIP1;4 and AtPIP2;4, but also used statistical methods to assess potential functional synergy between them, further highlighting their significance in plant growth and disease resistance. The investigation began with analyzing the impact of AtPIP1;4 and AtPIP2;4 on plant growth regulation. We compared the growth and fresh weight of WT, *atpip1;4*, *atpip2;4*, *atpip1;4atpip2;4*, *AtPIP1;4*OE, *AtPIP2;4*OE, and atpip1;4atpip2;4*dOE* plants, confirming that both AtPIP1;4 and AtPIP2;4 are positive regulators of plant growth and that they synergistically regulate the fresh weight of the plants (Figure 1 and Figure 2). These results strongly suggest the importance of AtPIP1;4 and AtPIP2;4 in plant growth.

H_2_O_2_ is the second messenger that mediates downstream immune reactions and plays a crucial role in regulating plant immune responses [1,2,3,53]. Our research demonstrates that AtPIP1;4 and AtPIP2;4 are concomitant channels for H_2_O_2_ transport, and they synergistically regulate plant growth and disease resistance (Figure 1, Figure 2, Figure 3, Figure 4, Figure 5, Figure 6 and Figure 7). H_2_O_2_ has been proven to be a substrate of AQPs, with many PIPs demonstrated to transport H_2_O_2_ in plants [8,9,10,11,29,43]. However, there is currently no evidence indicating whether PIPs work together in transporting H_2_O_2_. We have shown in previous studies that AtPIP1;4 can mediate H_2_O_2_ transmembrane transport, but H_2_O_2_ ectopic reduction was not completely eliminated in the *AtPIP1;4* mutant, indicating that there may be other PIPs in Arabidopsis that transport H_2_O_2_ together with AtPIP1;4 [9]. We used a fluorescence probe of H_2_O_2_ to examine the translocation of exogenously applied H_2_O_2_ to the cytoplasm. Compared with WT plants, the cytoplasmic H_2_O_2_ content significantly increased in *AtPIP1;4* and *AtPIP2;4* overexpression lines, and the double overexpression lines of *AtPIP1;4* and *AtPIP2;4* had an even higher cytoplasmic H_2_O_2_ content (Figure 3, Figure 4 and Figure 5). These collective findings demonstrate that AtPIP1;4 and AtPIP2;4 are common channels for the translocation of H_2_O_2_ from the apoplast to the cytoplasm.

In our previous study, we found that AtPIP1;4 acts as an immune-related facilitator in Arabidopsis, responsible for transporting H_2_O_2_ from the apoplast to the cytoplasm, leading to the activation of PTI and SAR, and resulting in plant resistance to pathogens [9]. In this study, we confirmed that AtPIP1;4 and AtPIP2;4 work together to transport pathogen-induced apoplastic H_2_O_2_ into the cytoplasm, and there is no significant difference in H_2_O_2_ production among different strains (Figure 6 and Figure S4). This indicates that AtPIP1;4 and AtPIP2;4 do not affect H_2_O_2_ production, but regulate plant defense by transporting H_2_O_2_, which is similar to earlier reports [9,10,11]. The entry of apoplastic H_2_O_2_ into the cell acts as a signaling molecule to activate downstream immune responses, such as callose deposition and defense gene expression. The apoplastic H_2_O_2_ into the cytoplasm acts as a signaling molecule to activate downstream immune responses, such as callose deposition and defense gene expression [53,54]. As expected, compared with WT plants, *AtPIP1;4* and *AtPIP2;4* overexpression lines showed stronger resistance to DC3000, and the double overexpression line of *AtPIP1;4* and *AtPIP2;4* exhibited the strongest resistance to the pathogen among all tested plants (Figure 7D,E). This suggests that the roles of AtPIP1;4 and AtPIP2;4 in plant immunity may be overlapping. We observed enhanced callose deposition in the *AtPIP1;4*OE and *AtPIP2;4*OE, which was further enhanced in *AtPIP1;4AtPIP2;4d*OE plants (Figure 7A,B). Compared with WT plants, the expression of *PR1* and *PR2* was significantly higher in *AtPIP1;4*OE and *AtPIP2;4*OE lines, and even higher in the AtPIP1;4 and AtPIP2;4 double overexpression line (Figure 7C). We also demonstrated that AtPIP1;4 and AtPIP2;4 are essential for the typical PAMP-triggered PTI activation (Figure 8). Compared with WT plants, flg22 and chitin-induced callose deposition was significantly suppressed in *atpip1;4atpip2;4* plants, and the expression of PTI marker genes *MPK3* and *FRK1* could not be properly induced in *atpip1;4atpip2;4* (Figure 8 and Figure S5). This indicates that flg22 and chitin-induced PTI responses require the cooperation of AtPIP1;4 and AtPIP2;4. In summary, our results strongly support that AtPIP1;4 and AtPIP2;4 work together to transport apoplastic H_2_O_2_ into the cytoplasm to regulate plant growth and disease resistance.

## 4. Conclusions

In summary, we have demonstrated that AtPIP1;4 and AtPIP2;4 function synergistically as concomitant channels for H_2_O_2_ transport, playing a pivotal role in both plant growth and the enhancement of disease resistance. Through the comparative analysis of growth phenotypes of wild-type, single and double mutants, and overexpressing lines, we found that AtPIP1;4 and AtPIP2;4 are positive regulators of plant growth. Furthermore, our findings elucidate the dual role of AtPIP1;4 and AtPIP2;4 in mediating H_2_O_2_ translocation from the apoplast to the cytoplasm, leading to increased callose deposition and enhanced defense gene expression to strengthen plant immunity. Future studies should focus on the detailed molecular mechanisms by which AtPIP1;4 and AtPIP2;4 interact with other components of the H_2_O_2_ signaling pathway, as well as their potential roles in response to abiotic stresses. This research highlights the significance of aquaporins in plant physiological and pathological processes, paving the way for future developments in agricultural biotechnology.

## 5. Materials and Methods

### 5.1. Plant Material and Growth Conditions

The Arabidopsis ecotypes *atpip1;4*, *atpip2;4*, *ATPIP1;4*OE, and *ATPIP2;4*OE were generated in the previously described Col-3 background [9,51]. The *atpip1;4atpip2;4* and *ATPIP1;4ATPIP2;4*dOE hybrid lines were generated in the HD laboratory and used for this study as F3 self-fertilized homozygotes. All gene constructs were sequenced to ensure their correctness. The primers used in this study are provided in Table S1. Seeds were germinated in plastic trays filled with nutrient soil and vermiculite (1:3). A total of 5 days later, the germinated seedlings were transferred to pots with the same substrate. Seed germination and plant growth were carried out in a growth chamber under 23 °C, 250 μM quanta/m_2_/s illumination, and an 8 h light/16 h dark photoperiod.

### 5.2. Bacterial Infection and Disease Assessment

Bacterial inoculation was performed on 30-day-old plants without any treatment. The *Pst* DC3000 inoculum was prepared as an aqueous bacterial suspension, adjusted to an optical density of 0.05 at 600 nm, with a final concentration of 10 mM MgCl_2_. The inoculum and mock control (10 mM MgCl_2_) were amended with 0.03% *v*/*v* Silwet L-77 and applied to the plants by spraying on the top of the plants. The bacterial count in plant leaves was measured at 72 h to assess the extent of DC3000 infection. At 9 dai, the extent of leaf chlorosis and necrosis symptoms were recorded by photographing the leaves. Differences in leaf DC3000 counts and the extent of leaf bacterial populations and symptom severity among different plant genotypes were used to assess the impact of AtPIP1;4 and AtPIP2;4 on immunity levels [9,44].

### 5.3. Plant Treatment

Aqueous solutions of 0.2 mM H_2_O_2_, 1 μM flg22 aqueous solution, and 0.1 mg/mL chitin solution were mixed with 0.03% *v*/*v* Silwet L-77. Each solution was individually sprayed on the top of 30-day-old plants. The top one-third of fully expanded leaves on the plants were used for H_2_O_2_ transport, callose deposition, and qRT-PCR.

### 5.4. H_2_O_2_ Transport Assay

The H_2_O_2_ transport assay was performed following the previously described method [9]. Briefly, plant leaves were infiltrated with 100 μM H_2_DCF, AR, or AUR dyes. Leaf samples were taken, avoiding the incision site, and observed under a CLSM. The fluorescence of H_2_DCF was captured using an excitation filter of 460–490 nm and an emission filter of 525 nm. The excitation filter and emission wavelengths for AR and AUR were 543 nm and 585–610 nm. The fluorescence densities in leaf discs were quantified with a SpectraMax M5 96-microplate luminometer (Molecular Devices, Silicon Valley, San Jose, CA, USA) to estimate relative levels of intracellular H_2_O_2_.

### 5.5. Callose Deposition Assay

Leaves were immersed in a solution consisting of 10 mL of phenol, glycerol, lactic acid, water, and 95% ethanol (1:1:1:1:1:2 *v*/*v*) for decolorization until the leaves became transparent. The leaves were then stained with aniline blue for 4 h in the dark. Leaf samples were observed under UV light at a wavelength of 340–380 nm using a Nikon microscope (Tokyo, Japan).

### 5.6. Gene Expression Analysis

Total RNA from leaves was extracted using the RNA-easy Isolation Reagent Kit (Vazyme, R701-01, Nanjing, China). Reverse transcription was performed on 1 μg of total RNA using the HiScript QRT Super Mix (Vazyme, R123-01, Nanjing, China). *AtActin* gene was used as the internal control gene, and qRT-PCR was carried out in a 20 µL reaction using ChamQ SYBR qPCR Master Mix (Vazyme, Q711, Nanjing, China). The primers for all qRT-PCR are listed in Table S1. The reactions were conducted on the Quant Studio 3 Real-Time PCR System (Applied Biosystems, Waltham, MA, USA) with the following conditions: 95 °C for 30 s, followed by 40 cycles of 95 °C for 10 s, 60 °C for 30 s, then 95 °C for 15 s, 60 °C for 1 min, and again 95 °C for 15 s to obtain the melting curve. The expression levels of each test gene relative to the constitutively expressed *AtActin* reference gene were determined by the 2^−ΔΔCt^ method [55].

### 5.7. SOD Activity Analysis

The SOD activity in the leaves was determined using the SOD Assay Kit (Beyotime S0103, Shanghai, China). Briefly, 0.2 g of leaves were harvested, ground into powder in liquid nitrogen, and resuspended in phosphate buffer. The suspension was centrifuged at 12,000× *g* for 5 min at 4 °C, and the supernatant was collected for measuring the SOD activity in the leaves.

### 5.8. Statistical Analysis

Quantitative data were analyzed using Student’s *t*-test or analysis of variance (ANOVA), followed by Duncan’s multiple range test, with GraphPad Prism 8.0.2 (https://www.graphpad.com/), accessed on 2 March 2023. The number of experimental replicates is specified in the figure legends.

## Data Availability

The datasets supporting the conclusions of this article are included within the article and Supplementary Materials.

## Supplementary Materials

The following supporting information can be downloaded at https://www.mdpi.com/article/10.3390/plants13071018/s1, Figure S1: Both AtPIP1;4 and AtPIP2;4 contribute to plant growth; Figure S2: AtPIP1;4 and AtPIP2;4 collaborate to transport H_2_O_2_ from apoplast to cytoplasm in Arabidopsis; Figure S3: Superoxide dismutase activities in plants treated with H_2_O_2_; Figure S4: Superoxide dismutase activities in plants treated with DC3000; Figure S5: AtPIP1;4 and AtPIP2;4 synergize in intensifying PTI. Table S1: Information on genes tested and primers used in this study.
