# Supplementary material for: AtPIP1;4 and AtPIP2;4 Cooperatively Mediate H2O2 Transport to Regulate Plant Growth and Disease Resistance
Source: Plants (Basel). 2024 Apr 3;13(7):1018. doi: 10.3390/plants13071018 (PMC11013698; doi:10.3390/plants13071018)
Supplement: Supplementary file 1 [file plants-13-01018-s001.zip › Supplementary Materials-R1.pdf]

AtPIP1;4 and AtPIP2;4 Are Concomitant Channels for H_2O_2 Transport Related to Plant Growth and Disease Resistance

Xiaohui Yao [1,†], Yanjie Mu [1,2,†], Liyuan Zhang [1], Lei Chen [1], Shenshen Zou [1], Xiaochen Chen [1], Kai Lu [1*], and Hansong Dong [1]

Supplementary material

Table S1 Information on genes tested and primers used in this study

Gene	Locus code (source)	Primers / product length (bp) / subjects
AtPIP1;4	AT4G00430	5'-GCCTTTCAAGAATATGTATTTGGG-3',<br>5'-CCGGAGCACTAATCACCTTAGAC-3' / qRT-PCR
		RP: 5'-CATGGTCTTTCTACAGAGCCG-3'<br>LP: 5'-AATGGTCCAACCCAGAAAATC-3'<br>LB: 5'-GCCTTTTCAGAAATGGATAAATAGCCTTGCTTCC-3' / Mutants validation
		5'-ATGGAAGGCAAAGAAGAAGATGTACG-3'<br>5'-CTTGTACAGCTCGTCCATGCCG-3' / Validation of gene overexpression plants
AtPIP2;4	AT5G60660	5'-TTAAAGCTCTTGGCTCATTTGG-3',<br>5'-CCACTTAATTCATTTGTAATCATCC-3' / qRT-PCR
		RP: 5'-ATACCCAATATTCCTACGCCG-3'<br>LP: 5'-AAATCAAGGGTCGACCAAAAC-3'<br>LB: 5'-TAGCATCTGAATTTCATAACCAATCTCGATACAC-3' / Mutants validation
		5'-ATGGCAAAAGACTTGGATGTGAACG-3'<br>5'-CTTGTACAGCTCGTCCATGCC-3' / Validation of gene overexpression plants
AtFRK1	AT2G19190	5'-CGGTCAGATTTCAACAGTTGTC-3',<br>5'-AATAGCAGGTTGGCCTGTAATC-3' / qRT-PCR
AtMPK3	AT3G45640	5'-CTCACAATGAGGATGCGAAA-3',<br>5'- TTCGGGTCGTGCAATTTAG-3' / qRT-PCR
AtActin	AT3G18780	5'-GGCTCCTCTTAACCCAAAGG-3',<br>5'-CCCTCGTAGATTGGCACAGT-3' / qRT-PCR
AtPR1	At2G14610	5'-TTCTTCCCTCGAAAGCTCAA-3',<br>5'- AAGGCCCACCAGAGTGTATG -3' / qRT-PCR
AtPR2	At3G57260	5'-ATTCGACGCAAATCTCGACT-3',<br>5'-ATAGCTTTCCCTGGCCTTCT-3' / qRT-PCR

Supplementary Figures

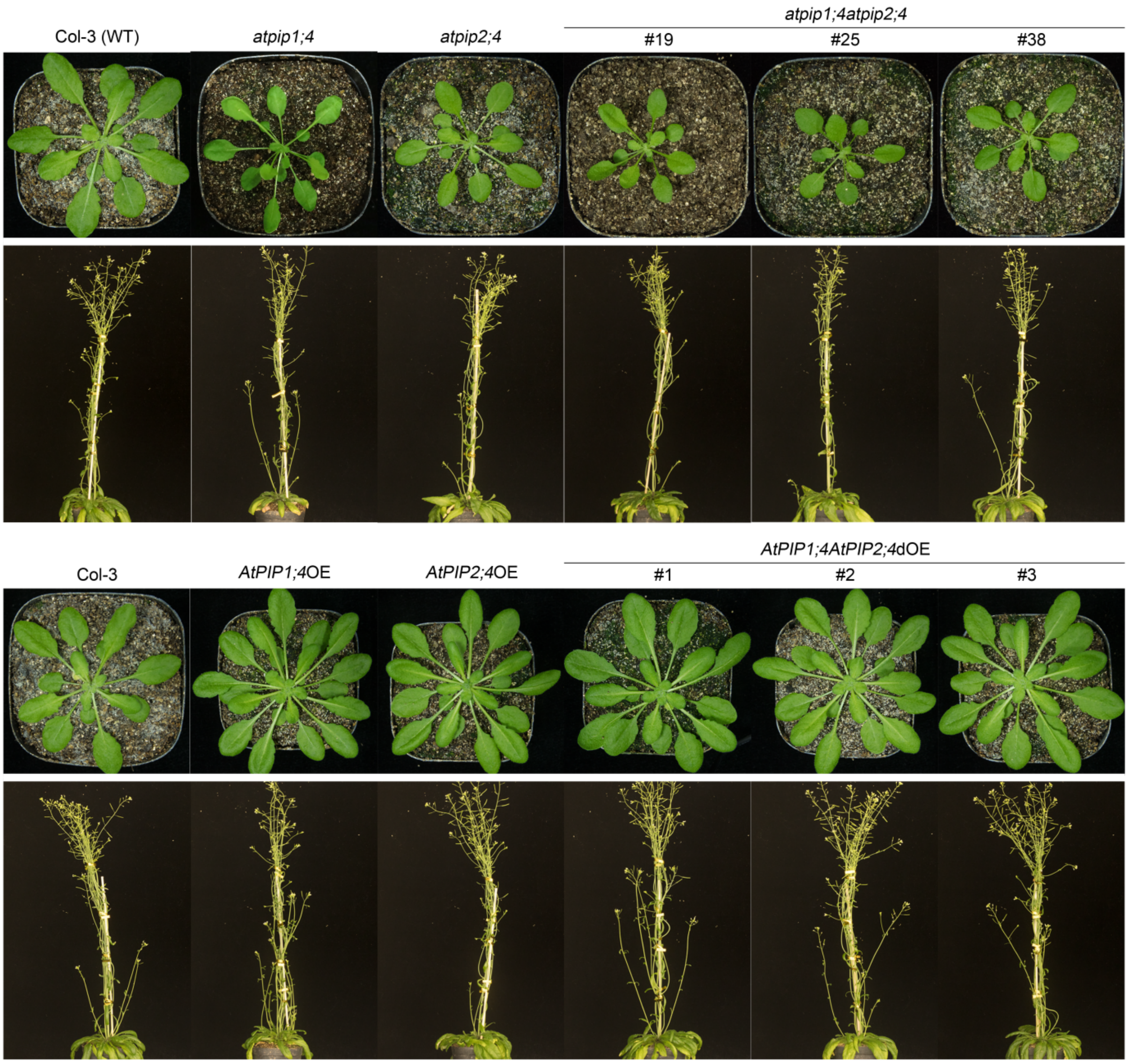

Figure S1. Both AtPIP1;4 and AtPIP2;4 contribute to plant growth, related to Figure 1. Photographs of 4- and 6-week-old Arabidopsis plants during growth, respectively.

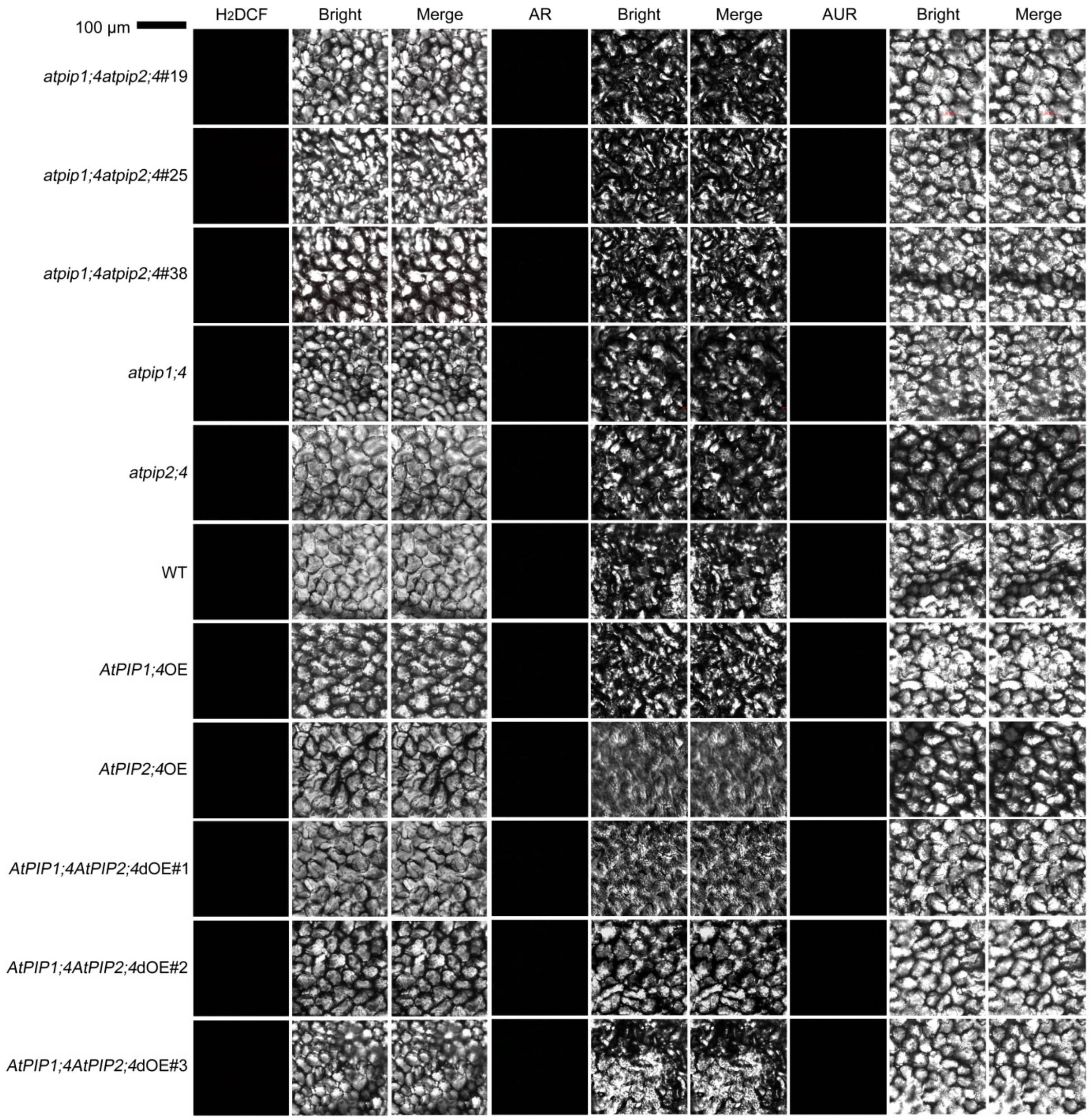

Figure S2. AtPIP1;4 and AtPIP2;4 collaborate to transport H_2O_2 from apoplast to cytoplasm in Arabidopsis, related to Figure 3. LSCM images showing H_2DCF-, AUR-probed apoplastic H_2O_2 and AR-probed cytoplasmic H_2O_2 in leaves of the Arabidopsis. The plants 45 minutes before LSCM had been treated with H_2O.

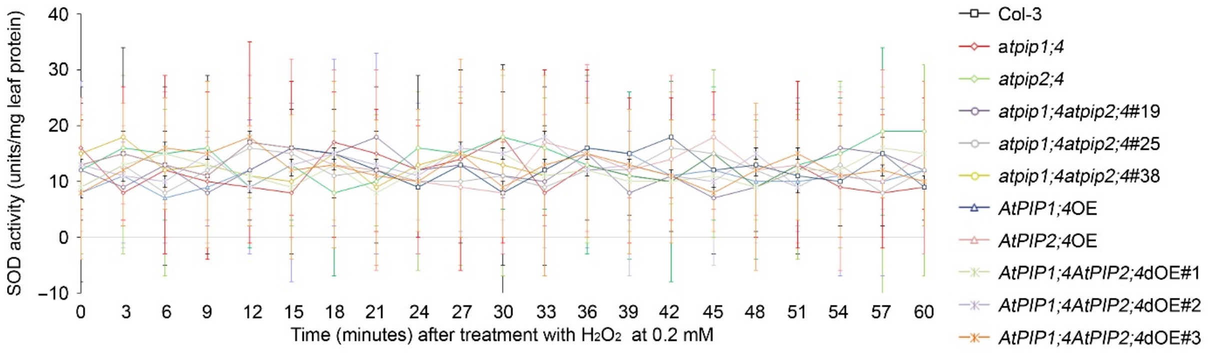

Figure S3. Superoxide dismutase activities in plants treated with H_2O_2, related to Figure 5. Chronological changes of superoxide dismutase (SOD) activity in leaves of plants in response to 0.2 mM H_2O_2. Data are shown as means ± SEM (n = 6).

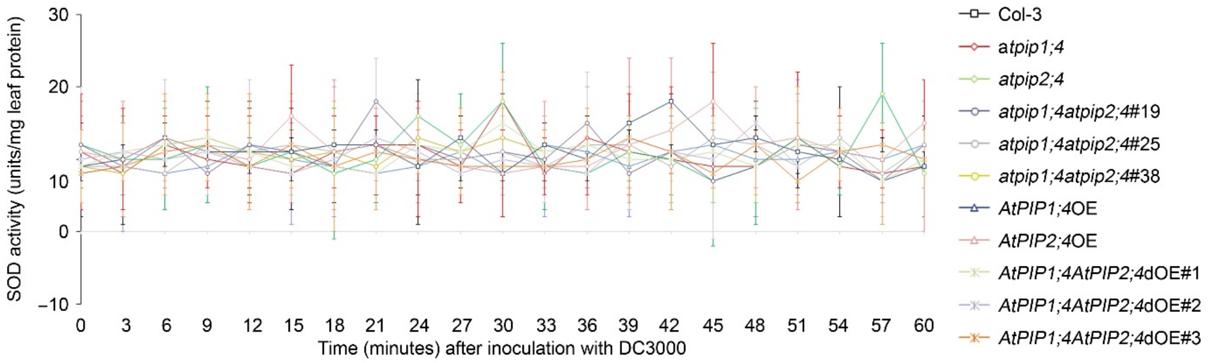

Figure S4. Superoxide dismutase activities in plants treated with DC3000, related to Figure 6. Chronological changes of superoxide dismutase (SOD) activity in leaves of plants in response to DC3000. Data are shown as means ± SEM (n = 6).

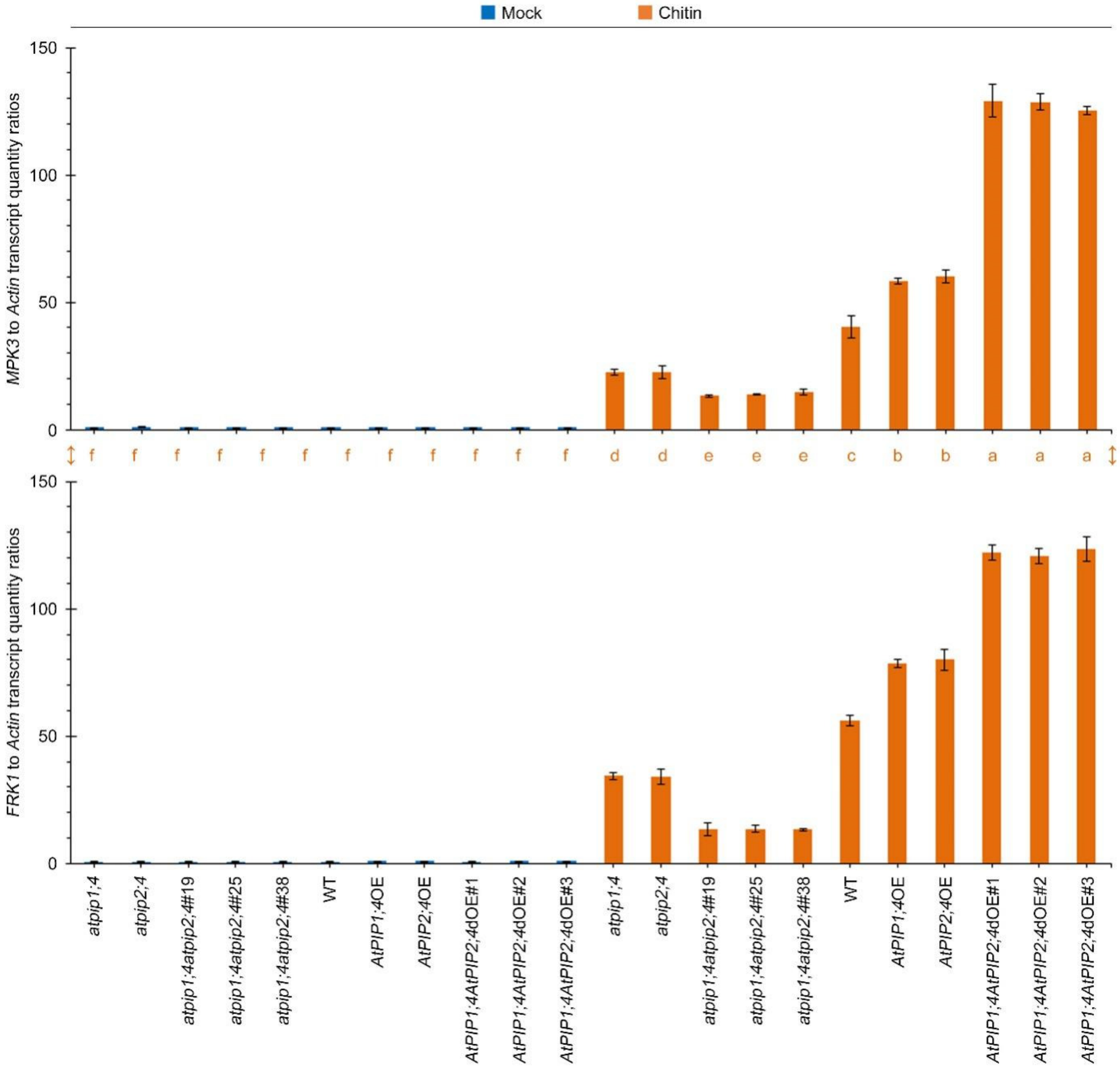

Figure S5. AtPIP1;4 and AtPIP2;4 synergize in intensifying PTI, related to Figure 8. Plants were treated with water (mock), an aqueous solution of 10 μM chitin and then used in the qRT-PCR assays. Expression levels of PTI-related genes *MPK3* and *FRK1* expression levels in plants treated with chitin for 60 min. Data were shown as means ± SEM ($n = 6$). Lowercase letters indicate significant differences by one-way ANOVA and Duncan's multiple range tests ($P < 0.05$).
